# 16S rRNA metagenomic dataset on endophytic bacterial community of the cashew plant (*Anacardium occidentale* L.) grown in Dak Lak Province of Vietnam

**DOI:** 10.1016/j.dib.2024.110039

**Published:** 2024-01-09

**Authors:** Dinh Minh Tran, Thi Huyen Nguyen

**Affiliations:** Institute of Biotechnology and Environment, Tay Nguyen University, Buon Ma Thuot, Dak Lak 630000, Vietnam

**Keywords:** Cashew endophytic microbiome, 16S rRNA metagenomics, Proteobacteria, Biosynthesis

## Abstract

Vietnam is currently one of the largest producers and exporters of cashew nuts in the world. Cashew (*Anacardium occidentale* L.) is one of the main industrial crops cultivated in Dak Lak Province of Vietnam. Comprehending the endophytic bacteria of this plant, a new biofertilizer for sustainable cashew nut production can be progressed. In this report, the cashew root sample was collected from cashew fields in 2021 in Dak Lak. The DNeasy Powersoil kit was used to extract the genomic DNA of endophytic bacteria from the root sample. The 16S rRNA genes (V1–V9 regions) were amplified by PCR, and libraries of amplicons were prepared using the Swift amplicon 16S plus ITS panel kit. The Illumina MiSeq platform was applied to sequence amplicon libraries using 16S rRNA metagenomics. Taxonomic analyses showed that Gammaproteobacteria (38.77 %) and Alphaproteobacteria (37.76 %) were the predominant classes among the endophytic bacteria. Functional analyses revealed that biosynthesis (72.78 %) was the primary function of the endophytic bacterial community. Raw sequences (Fastq files) have been deposited in Mendeley Data [1]. The obtained data provide insight into the endophytic bacterial community of cashews cultivated in Dak Lak Province of Vietnam. The data are valuable for further developing a new biofertilizer for cashew nut production using endophytic bacteria. Ours is the first report about endophytic bacterial communities of cashews cultivated in this province as well as the Central Highlands of Vietnam.

Specifications Table

**Table:** 

Subject	Microbiology: Microbiome
Specific subject area	Metagenomics, Molecular biology, Bioinformatics
Data format	Raw, Filtered, and Analyzed
Type of data	Figures, Fastq files
Data collection	- Collected the root sample of cashew - Extracted the microbial metagenomic DNA - Conducted metagenomic sequencing - Analyzed metagenomic sequences
Data source location	• Institution: Institute of Biotechnology and Environment, Tay Nguyen University • Commune/District/Province/Region: Hoa Phu/Buon Ma Thuot/Dak Lak/The Central Highlands • Country: Vietnam • Latitude and longitude coordinates for collected samples: 12°35′08′′N, 107°56′09′′E; 12°35′13′′N, 107°56′10′′E; 12°35′14′′N, 107°56′20′′E; 12°35′15′′N, 107°56′26′′E; 12°34′51′′N, 107°56′28′′E
Data accessibility	Raw sequences (Fastq files) Repository name: Mendeley Data Data identification number: doi: 10.17632/gtx4534dtx.1 Direct URL to data: https://data.mendeley.com/datasets/gtx4534dtx/1

## 1 Value of the Data

• Data provide taxonomic and functional profiles of endophytic bacteria of the cashew plant cultivated in Dak Lak Province, Vietnam.
• Data can be valuable for comparing endophytic bacteria of the cashew plant grown in Dak Lak and others.
• Data can be valuable for developing a new biofertilizer for sustainable cashew nut production based on endophytic bacteria.

## 2 Background

Vietnam is one of the world's top 10 producers and exporters of cashew nuts from 2011 to 2022. Cashew is one of the main perennial industrial crops cultivated in the Central Highlands region of Vietnam. Vietnam had 322,300 hectares of cashew planted and produced 341,700 tons in 2022, in which the Central Highlands contributed 83,900 hectares and 33,560 tons, respectively. Among 5 provinces in this region, Dak Lak was the biggest producer of cashew nuts [2]. Currently, chemical fertilizers are usually used for cashew nuts production in the province. However, it is clear that chemical fertilizers can impact the environment badly, reduce cashew nuts' quality, and increase farmers' input. Hence, indigenous bacteria are thought to be the best strategy to produce sustainably cashews. Data on endophytic and rhizospheric bacteria of black pepper, coffee, and sugarcane plants cultivated in Dak Lak have been explored to develop new cultivation techniques for the sustainable production of these crops [3], [4], [5], [6]; however, to the best of our knowledge, no data on the endophytic microorganisms of the cashew plant growth in the province as well as in the Central Highlands have been reported. This work aimed to establish a dataset on the endophytic bacteria of the cashew plant grown in Dak Lak Province, the Central Highlands of Vietnam.

## 3 Data Description

**Fig. 1:**
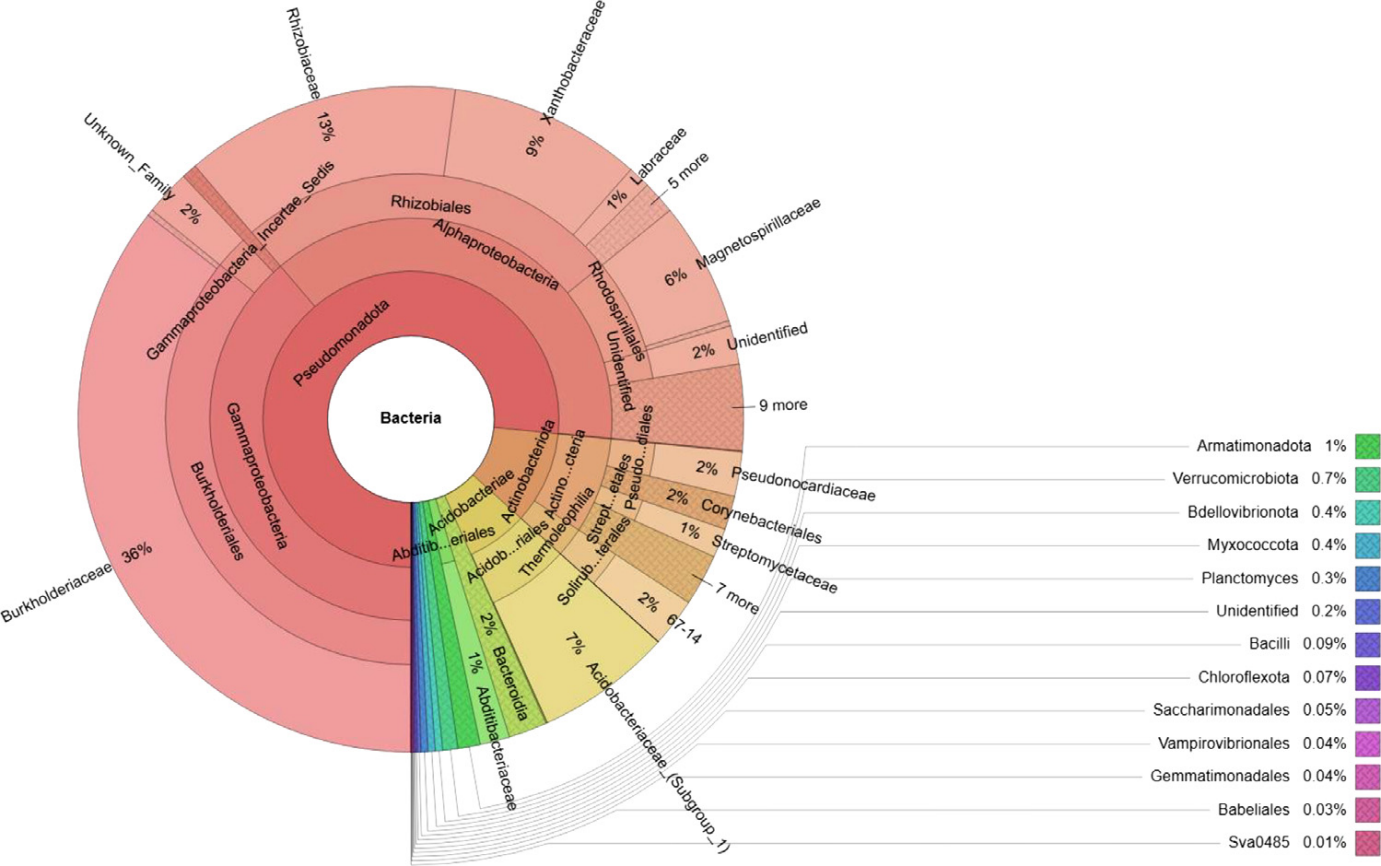
Krona chart representation of taxonomic classification of the cashew root endophytic bacteria.

In the current report, 253,461 reads were used for analysis after quality filtering of 287,398 raw reads. Fig. 1 shows that 17 bacterial phyla were detected from the root sample of cashew collected from Dak Lak Province. Of these phyla, Pseudomonadota (76.57 %) was the most predominant, followed by Actinobacteriota (10.1 %), Acidobacteriota (6.68 %), Bacteroidota (1.86 %), Abditibacteriota (1.44 %), and Armatimonadota (1.08 %). A total of 26 classes were assigned from these phyla. Among them, Gammaproteobacteria took 38.77 %, Alphaproteobacteria accounted for 37.76 %, Actinobacteria for 7.72 %, Acidobacteriae for 6.68 %, Thermoleophilia for 2.38 %, Bacteroidia for 1.86 %, and Abditibacteria for 1.44 %. A total of 64 orders were identified from the classes. Of these classes, Burkholderiales was the most predominant (35.82 %), followed by Rhizobiales (25.44 %), Acidobacteriales (6.63 %), Rhodospirillales (6.27 %), Solirubrobacterales (2.37 %), Gammaproteobacteria (2.2 %), Pseudonocardiales (2.16 %), Corynebacteriales (1.64 %), Abditibacteriales (1.44 %), Streptomycetales (1.41 %), Chitinophagales (1.18 %), and Dongiales (1.07 %). Additionally, from the orders, 86 families were detected. Burkholderiaceae (35.51 %) was the primary family, followed by Rhizobiaceae (13.37 %), Xanthobacteraceae (9.36 %), Acidobacteriaceae (6.61 %), Magnetospirillaceae (6.01 %), Pseudonocardiaceae (2.16 %), Abditibacteriaceae (1.44 %), and Chitinophagaceae (1.18 %). Finally, 103 genera were identified from these families. Raw sequences (Fastq format) from this work have been uploaded to Mendeley Data and can be accessed at https://data.mendeley.com/datasets/gtx4534dtx/1.

**Fig. 2:**
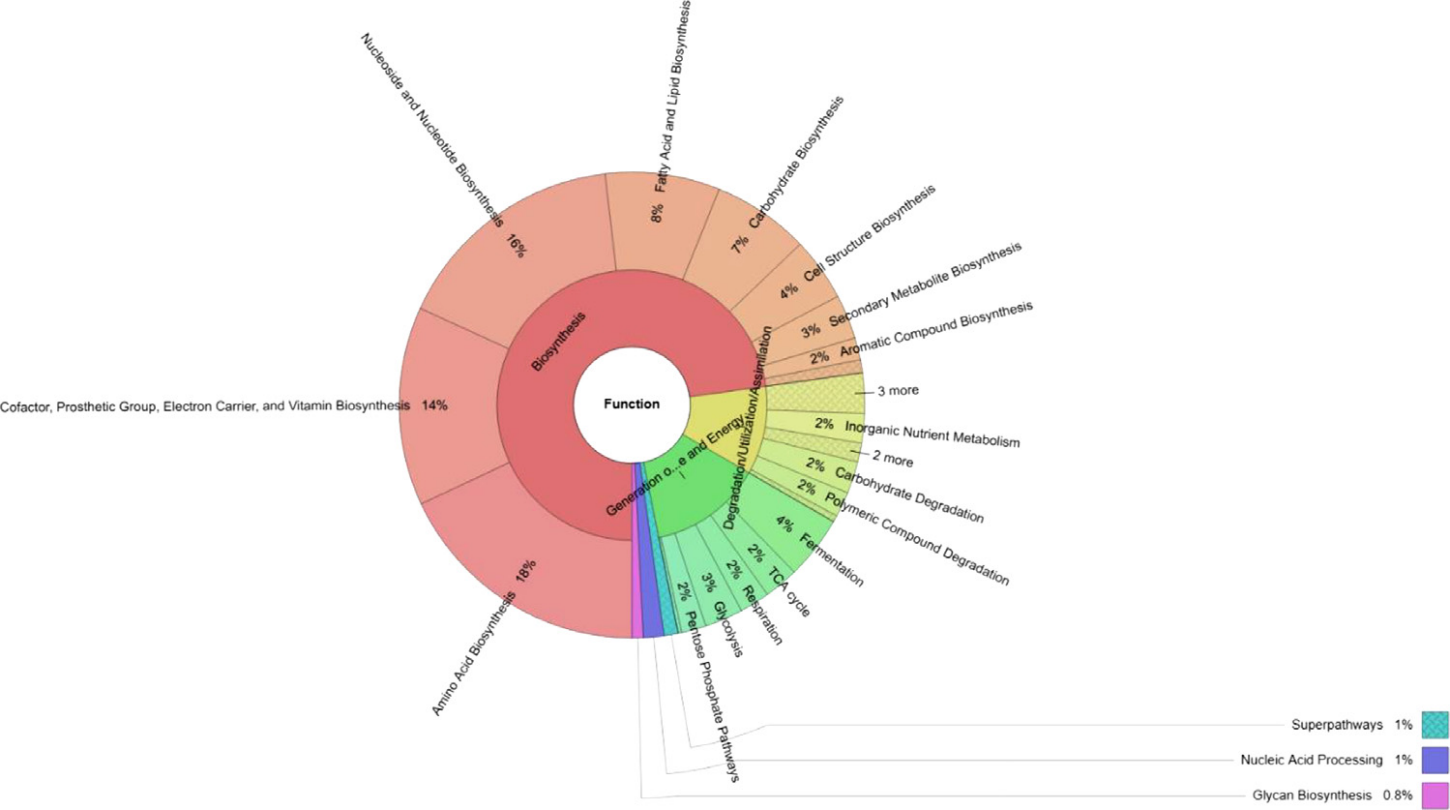
Krona chart representation of functional profiles of the cashew root endophytic bacteria.

Fig. 2 shows that biosynthesis (72.78 %) was the primary function of the cashew endophytic bacteria, followed by the generation of precursor metabolites and energy (13.48 %), and degradation/utilization/assimilation (10.56 %). Of the functions involved in biosynthesis, amino acid biosynthesis (18.03 %) was the most abundant, followed by nucleoside and nucleotide biosynthesis (16.34 %); cofactor, prosthetic group, electron carrier, and vitamin biosynthesis (13.79 %); fatty acid and lipid biosynthesis (7.96 %); carbohydrate biosynthesis (6.74 %); cell structure biosynthesis (4.41 %); secondary metabolite biosynthesis (3.08 %); and aromatic compound biosynthesis (1.53 %). Raw sequences (Fastq format) from this work have been uploaded to Mendeley Data and can be accessed at https://data.mendeley.com/datasets/gtx4534dtx/1.

## 4 Experimental Design, Materials and Methods

### 4.1 Sampling

This work collected five cashew root samples (5 to 30 cm under the soil surface) from 5 cashew fields on 30 October 2021 in Hoa Phu Commune, Buon Ma Thuot City, Dak Lak Province of Vietnam. The samples were then combined to create a representative sample. The root sampling, treatment, and storage were conducted as described by Tran et al. [6].

### 4.2 Genomic DNA extraction, library preparation, and sequencing

Genomic DNA extraction, library preparation, and sequencing were conducted as described previously [3], [4], [5], [6]. Briefly, metagenomic DNA was extracted from 0.3 g of the root sample using the DNeasy PowerSoil kit (Qiagen, USA). The 16S rRNA gene amplicon sequencing libraries were prepared using the Swift amplicon 16S plus internal transcribed spacer panel (Swift Biosciences, USA). The libraries were then sequenced using the Illumina MiSeq platform (2  ×  150 PE).

### 4.3 Analysis of data

Adapters, primers, and low-quality sequences (average score <20 and read length <100 bp) were removed using Trimmomatic v.0.39 [7] and Cutadapt v.2.10 [8]. Reads were clustered and dereplicated into amplicon sequence variants using the q2-dada2 plugin and the QIIME2 pipeline version qiime2-2020.8 [9]. Taxonomy profiles were analyzed using QIIME2 against the SILVA database [10]. After analysis, the phyla names of prokaryotes were re-updated, as demonstrated by Oren and Garrity [11]. Functional profiles were deduced using PICRUSt2 v.2.3.0-b [12] and the MetaCyc database [13].

## Limitations

Not applicable.

## Ethics Statement

The current work does not involve human subjects, animal experiments, or any data collected from social media platforms.

## CRediT Author Statement

**Dinh Minh Tran:** Conceptualization, Methodology, Investigation, Formal analysis, Software, Data curation, Validation, Visualization, Writing review & editing; **Thi Huyen Nguyen:** Sampling, Investigation, Formal analysis.

## Data Availability

Dataset on endophytic bacterial communities of the cashew plant grown in Dak Lak, Vietnam (Original data) (Research Data)
